# Physiological and Therapeutical Action of Ergot

**Published:** 1882-04

**Authors:** 


### THE PHYSIOLOGICAL AND THERAPEUTICAL ACTION OF ERGOT.

In the March number of the New York *Medical Journal and Obstetrical Review,* Dr. Etienne Evetzky, of New York, concludes the publication of his Joseph Mather Smith prize essay on ergot. Although dealing mainly with the physiological and therapeutical actions of the drug, the author gives a comprehensive account of the history of the different varieties of ergot, their botanical relations, their microscopical structure, and their chemical composition ; the methods of their production, collection, preservation, and preparation for medicinal use ; the relations of ergot to other remedies, etc. In comparing the action of ergot with that of a number of other excito-motors of the organic muscular tissue, an arbitrary group of which, the author thinks, ergot may be taken as the typical representative, he remarks that strychnia is most closely allied to ergot in its effects, the main difference being that strychnia acts with far greater energy on the spinal motor centres of the voluntary muscular tissue. Digitalis is distinguished by its predominant stimulating action on the heart. The chief difference between the action of ergot and that of Calabar bean lies in the early occurrence of a paretic state of the voluntary motor apparatus after doses of the latter drug that are not quite toxic. Atropia and nitrite of amyl are mentioned as antagonists to ergot. For hypodermic administration we may use the extract, the fluid extract, or sclerotic acid, diluted in water, with or without the addition of glycerine or alcohol, which latter substances, the author thinks, do not improve the solution in the least. The solution should always be clear and not too old, and should be made somewhat alkaline if the injections are particularly painful. The solution should invariably be injected into the muscular tissue, and it is well to begin with small doses. The therapeutical applications of ergot are considered under five heads: 1. Disorders of the circulation and diseases of the organs of circulation. 2. Paretic conditions of the organs composed of organic muscular tissue, the circulatory system excepted. 3. Inflammatory and other morbid enlargements and growths. 4. Abnormal secretions. 5. Symptoms referable to the nervous system, and depending chiefly upon circulatory disorders within it. In regard to contraindications to the use of ergot, it should be used with extreme caution in patients with an enfeebled heart. Pregnancy is not an absolute contraindication. The use of the drug should be suspended during menstruation, unless it is given for some special condition of that function. To avoid disturbing the digestion it is best to give the drug by the rectum or hypodermically. The remainder of the article deals with the special diseases in which ergot seems capable of effecting good results.

